# Longitudinal Association of Executive Function and Balance in Community-Dwelling Older Adults

**DOI:** 10.1093/geroni/igab046.2608

**Published:** 2021-12-17

**Authors:** Jennifer Blackwood, Reza Amini, Gerry Conti, Quinn Hanses, Rebekah Taylor, Rachelle Naimi, Deena Fayyad

**Affiliations:** 1 University of Michigan--Flint, Michigan, United States; 2 University of Michigan-Flint, Flint, Michigan, United States; 3 University of Michigan - Flint, University of Michigan-Flint, Michigan, United States; 4 University of Michigan - Flint, Flint, Michigan, United States; 5 University of Michigan-Ann Arbor, Ann Arbor, Michigan, United States

## Abstract

Declines in Executive Function (EF) are associated with balance in community-dwelling older adults with Mild Cognitive Impairment (MCI). While this has been examined in cross-sectional studies, no longitudinal studies describe change over time. The purpose of this study was to examine how performance on the components of the Short Physical Performance Battery (SPPB) are associated with EF in community-dwelling older adults who transition into MCI. This secondary data analysis employed eight years of data from the National Health and Aging Trends Study dataset (2011 – 2018) with 1,225 participants in all eight waves (balanced). EF was measured with the Clock Drawing Test and SPPB balance tests included side-by-side, semi-tandem, full tandem, and single leg stance with eyes open or closed. Longitudinal ordered logistic regression was used to examine associations between each balance measure and EF while controlling for comorbidity, function, depression, gender, age, and ethnicity. EF was significantly associated with tandem, semi-tandem, and single leg stance after controlling for covariates. One point increase in SPPB can reduce the risk of EF impairment by 8.2% (Odds Ratio (OR)=0.918, p<0.001). Among SPPB components, semi-tandem (OR=0.468) and side-by-side (OR=0.472) were the strongest predictors of EF impairment. Declines in both EF and balance performance occurred over an eight-year period in adults. This may reflect common neural processes shared between the cognitive and motor areas of the central nervous system. Best practice suggests screening both balance (tandem, semi-tandem, or single leg stance) and EF in the clinical assessment of community-dwelling older adults.

